# Zika Virus Infection in Asymptomatic Pregnant Women

**DOI:** 10.3390/idr17010002

**Published:** 2025-01-07

**Authors:** Kathia Guardado, Nayali López-Balderas, Jaime Morales-Romero, Clara Luz Sampieri, Roberto Zenteno-Cuevas, María Teresa Álvarez-Bañuelos, Ángel Ramos-Ligonio, María Cristina Ortiz-León, Miguel Varela-Cardoso, Hilda Montero

**Affiliations:** 1 Virus-Cell Interactions Laboratory, Institut Pasteur of Montevideo, Montevideo 11400, Uruguay; kaguilar@pasteur.edu.uy; 2 Biomedical Research Center, University of Veracruz, Xalapa 91190, Mexico; 3 Institute of Forensic Medicine, University of Veracruz, Veracruz 94294, Mexico; nayalopez@uv.mx; 4 Institute of Public Health, University of Veracruz, Xalapa 91190, Mexico; jamorales@uv.mx (J.M.-R.); csampieri@uv.mx (C.L.S.); rzenteno@uv.mx (R.Z.-C.); talvarez@uv.mx (M.T.Á.-B.); cortiz@uv.mx (M.C.O.-L.); 5 Faculty of Chemical Sciences, University of Veracruz, Orizaba 94340, Mexico; angramos@uv.mx; 6 Faculty of Medicine, University of Veracruz, Ciudad Mendoza 94740, Mexico; mvarela@uv.mx

**Keywords:** Zika virus, pregnancy, asymptomatic infection, newborn, viremia

## Abstract

**Background/Objectives**: Zika disease is caused by the Zika virus (ZIKV) and represents a major public health problem because of the complications in newborn babies from mothers who were infected during pregnancy. It is estimated that 80% of infected pregnant women are asymptomatic, which complicates the identification of infected individuals. In this study, we aimed to detect ZIKV in asymptomatic pregnant women and the effects in the newborns were analyzed. **Methods**: The presence of ZIKV was evaluated through endpoint reverse transcription–polymerase chain reaction (RT-PCR) in 114 blood samples from pregnant women treated at two hospitals in the state of Veracruz, Mexico. There was a follow-up of the participants until the birth of their newborns. **Results**: ZIKV RNA was detected in 4.4% (*n* = 5) of cases. In two positive cases, two consecutive samples were obtained, and one case of persistence of ZIKV in serum after 90 days after delivery was identified. A total of 80% of the positive cases were identified after the third trimester of pregnancy and 20% after the second trimester. Although ZIKV was shown to be a risk factor for low weight and low size at birth and prematurity, after adjustment for other variables, it did not show a significant association. In contrast, preeclampsia/eclampsia was identified as a significant risk factor for low birth weight. **Conclusions**: The prevalence of ZIKV found in this study suggests a latent circulation of this virus and highlights the importance of epidemiological surveillance in endemic zones. The prolonged viremia that was found suggests the need for more research because of the high impact which can mean the possible dissemination of the virus to the vector.

## 1. Introduction

Zika disease is caused by the ZIKV. This is a virus that belongs to the Flaviviridae family under the Flavivirus genus, with an RNA genome. The ZIKV was isolated for the first time in the Rhesus macaque from the forest of Zika, Uganda, in 1947 [1]. In the acute phase of Zika disease, the clinical manifestations last around 7 days and are mainly characterized by fever, arthralgia, headache, conjunctivitis, and exanthema [2]. It is estimated that 80% of the infected persons with ZIKV are asymptomatic [3]. Between the years 2016 and 2024 confirmed cases of ZIKV have been reported in México by the Mexican authorities in 29 of 32 states of the country in which the vector is distributed [4], showing 13,034 accumulated confirmed cases, of which 7156 are in pregnant women [5].

In 2015, ZIKV gained relevance in public health because of its association with complications in newborn babies whose mothers were infected during the pregnancy. Initially, microcephaly was considered a teratogenic sequel of ZIKV, but nowadays, it is well known that intrauterine infection by ZIKV causes a series of traits that have been grouped in a newly denominated syndrome called Congenital Zika Syndrome (CZS) [6]. Other growth alterations have been associated with CZS, like low weight, low height, and prematurity at birth due to intrauterine growth restriction during gestation [7,8]. The CZS is characterized by neurological alterations, arthrogryposis, and ophthalmic anomalies. More recently, hydrocephalus and postnatal microcephalus have been identified as possible traits of CZS [9]. A recent study carried out on children who had been exposed to ZIKV in utero but who did not present any CZS at birth showed alterations in their neurological development during their first 18 months after birth [10].

One major trait in pregnant women who were infected by ZIKV of high epidemiological impact is a potentially prolonged viremia with the possibility of passing the virus to the vector. In animal models with pregnant female Rhesus macaques, the viremia lasted from 28 to 70 days [11]. A similar observation was found in pregnant mothers infected with ZIKV [12], in whom the viremia lasted up to 107 days in the case of symptomatic women [13] and up to 70 to 72 days in the case of asymptomatic women [14]. Pregnant mothers are a vulnerable group to ZIKV infection; however, in the case of asymptomatic individuals, the lack of signs and symptoms complicates the diagnosis and the follow-up by health authorities. Therefore, it is then of high importance that relevant studies are made to know the effects of prenatal infection by ZIKV in this demographical group. This study aims to determine the infection by ZIKV in a group of asymptomatic pregnant women in the state of Veracruz, Mexico, in 2019, three years after the 2016 breakout, as well as to create knowledge on the effects and complications for the newborn associated with the prenatal infection by this virus in the mother.

## 2. Materials and Methods

### 2.1. Study Design

This study was designed in two phases: phase 1 was a cross-sectional study to observe the prevalence of ZIKV in asymptomatic pregnant women, and phase 2 was a prospective cohort study with the finality of determining the effect of the prenatal infection by ZIKV in newborns of asymptomatic mothers. The study was performed from February to June of 2019 in the state of Veracruz, Mexico. This urban area is located in the Gulf of Mexico and has presented the highest incidence of confirmed cases of CZS between 2016 and 2018 [15].

### 2.2. Protocol Description

This study was approved by the Ethics and Research Committee of the High Specialty Hospital of Veracruz (HSHV) and by the Health Secretary. A screening was performed to detect positive cases of ZIKV in asymptomatic pregnant patients in the General Hospital of Tarimoya (GHT) and in the HSHV. A total of 114 samples were selected through a sampling of consecutive patients recruited after the required informed consents were granted. The inclusion criteria were women in the gestation period who requested services from the clinical analysis laboratory, diagnosis appointments, hospitalization, or emergency rooms without symptomatology referent to arbovirus during pregnancy and who agreed to participate in the research protocol. In the 114 patients included in this study, the newborns were followed through the clinical file.

### 2.3. Sample Processing and Viral RNA Extraction

The blood samples were obtained using extraction tubes BD Vacutainer^®^ (EDTA, BD, Franklin Lakes, NJ, USA) for serum with coagulation activator. The serum was obtained through centrifugation at 3500 rpm and stored at −80 °C until its processing. The viral RNA was extracted using a QIAamp Viral RNA mini kit (QIAmp, QIAGEN, Hilden, Germany), after which a diagnostic test was performed with an endpoint RT-PCR using the One-Step kit RT-PCR (QIAGEN, Hilden, Germany) according to the manufacturer’s directions. The primers used for the detection of the E protein of ZIKV were primFor GCTGGGGCAGACACCGGAAC and primRev GTCCACCGCCATCTGGACTG, and plasmids as positive controls. DENV and CHIKV detection were included as negative controls. A second blood sample was taken from two patients to determine the duration of the viremia: one of whom had her second sample taken after 15 days of the first sample and who was in her third trimester of pregnancy when both samples were taken; the second of these patients had her first sample taken prior to delivery and the second sample 90 days later.

### 2.4. Clinical Data Collection

The remaining variables of interest were collected using an instrument developed by the research team. This instrument comprises anthropometric measures and descriptive variables of the mother and the newborn that are part of the study. These variables are as follows:

- Cross-sectional design: main measures.
- Positive cases: patients with blood samples positive for ZIKV by endpoint RT-PCR.
- Negative cases: patients with blood samples negative for ZIKV by endpoint RT-PCR.
- Cohort study: main measures.
- Exposure: Asymptomatic pregnant woman positive for ZIKV by endpoint RT-PCR.
- Outcome measures: newborn characteristics.
- Underweight newborns: using the criteria given by the World Health Organization (WHO) and by the National Federation of Neonatology of Mexico (non-profit), it was established that underweight for a newborn is below 2500 g.
- Short height newborns: it was established following the parameters of Fenton grow chart provided by WHO as part of the vigilance measures in areas with high risk of ZIKV dissemination.
- Premature birth: using the criteria given by WHO and by the National Federation of Neonatology of Mexico (non-profit), it was established that a newborn is premature at less than 37 weeks.
- Abnormal cephalic circumference in newborns: it was established in a newborn according to gestational age when cephalic circumference was above or below the normal range, and it was determined following the parameters of the Fenton grow chart provided by WHO as part of the vigilance measures in areas with high risk of ZIKV dissemination.

### 2.5. Statistical Analysis

The prevalence of ZIKV in asymptomatic pregnant women was estimated after dividing the positive cases by the total number of pregnant women who received a prenatal screening test. The confidence intervals for proportions were calculated through a binomial exact test. The comparison between categorical variables was performed by a chi-square test or Fisher’s exact test whenever it was necessary. The probability for the newborns to present the listed abnormalities at birth was calculated through relative risk and confidence intervals of 95%. Odds ratios and confidence intervals at 95 % were calculated in a multivariate binary logistic regression analysis to adjust for the association between several outcomes (low birth weight, low birth length, and prematurity) with maternal asymptomatic ZIKV infection. Adjustment variables that could be confounding factors were maternal obstetric risk age and the presence of preeclampsia or eclampsia during pregnancy. The statistical significance was defined as a value of *p* ≤ 0.05. The statistical analysis was performed with SPSS (IBM SPSS Statistics 210; SPSS Inc., New York, NY, USA) and StatCalc (Epi Info; CDC, Atlanta, GA, USA).

## 3. Results

A total of 114 samples from pregnant women were studied. The patients were divided into two groups: positive and negative cases for ZIKV. All patients were negative for DENV and CHIKV. All the women in the first group were Mexican citizens with a mean age of 25.4 years, a mean weight of 77.4 kg, and a mean height of 162.7 cm. In the second group, 107 women were Mexican nationals, 1 Honduran national, and 1 Colombian national, with a mean age of 24.9 years, a mean weight of 72.12 kg, and a mean height of 156.06 cm. There was an abortion in the second group (Table 1). Despite there being women of other nationalities in the study, all of them resided in the state of Veracruz.

The prevalence of ZIKV in asymptomatic pregnant women (*n* = 114) was 4.4% (95% C.I., 1.4–9.9%). For GHT, of the total samples (*n* = 88), 3.4% (95% C.I., 0.7–9.6%) were positive for ZIKV. For HSHV, of the total samples (*n* = 26), 7.7% (95% C.I., 0.9–25.1%) were positive for ZIKV (Table 2).

To measure the effects of the maternal infection of ZIKV in newborns, a follow-up of the patients was performed until childbirth using access to the medical files. With the obtained data, the characteristics of the newborns were compared between the ZIKV-positive and the ZIKV-negative cases. The results show that 50% of the studied newborns from ZIKV-positive pregnant women presented either underweight or short height or were premature. The relative risk of developing underweight at birth was 6.4 (95% C.I., 1.6–24.8); 4.0 (95% C.I., 1.2–13.7) for short height and 3.6 (95% C.I., 1.1–12.1) for premature birth. Although the identified relative risks were statistically significant, there was no consistency in the findings after the analysis of categorical data using Fisher’s exact test for small samples (*p* > 0.05). There was no newborn from the exposed group who presented an altered cephalic perimeter at birth (Table 3).

Table 4 presents the statistically associated variables, adjusted in a multivariate analysis. The adjustment variables were maternal obstetric risk age and preeclampsia or eclampsia during pregnancy. In the simple model, a positive RT-PCR ZIKV result was associated as a risk factor for low birth weight (*p* = 0.03). However, when maternal obstetric risk age and preeclampsia/eclampsia variables were introduced in models 2 and 3, the positive RT-PCR ZIKV was shown to be a non-significant risk factor of all outcome variables.

In reference to the trimester of the pregnancy in which the first blood sample was collected, the results did not show any positive case during the first trimester. The third trimester had the highest number of cases (Table 5). Among the two patients with two blood samples, prolonged viremia was detected only in a patient from whom the sample was obtained in the moment of labor. This viremia had a duration of 90 days, and the patient presented cardiac issues, respiratory problems, and fatigue.

## 4. Discussion

ZIKV is currently circulating in Mexico as well as in other countries. The prevalence of infection by ZIKV in asymptomatic pregnant women varies worldwide because of the heterogeneity of the studied population, the time in which the study is performed, and host factors, including socioeconomic variables, climate and geographic factors, and vector ecology [16,17]. In Peru and Brazil, studies similar to our work showed a prevalence of 3.2% and 4.6%, respectively [18,19]. Of note, our study is the first of its type to be performed in Mexico, which showed that 4.4% of the sampled asymptomatic women were infected with ZIKV when the test was performed. ZIKV is a public health problem in Mexico since, according to the Mexican authorities, 54.9% of the ZIKV confirmed cases from 2015 to 2024 are in symptomatic pregnant women with Zika disease [5]. This shows the constant circulation of this arbovirus in endemic areas, positioning ZIKV as a continuous public health problem that must be addressed.

The ZIKV has three mechanisms of transmission, namely vertical, sexual, and by vector, and it generates persistence and is teratogenic [20,21]. Because the circulation of this virus is recent, the sequels of the infection are not yet completely known.

Pregnant women infected with ZIKV are at risk for developing adverse perinatal outcomes, including stillbirth, transplacental (vertical) viral transmission, neurodevelopmental disorders, and malformation syndromes, including congenital Zika syndrome (CZS) [22]. However, not all newborns of mothers infected with ZIKV during pregnancy show effects derived from it [23], which suggests the existence of protective factors that need to be known to design control guidelines and protect the mothers. Some newborns of mothers who were infected with ZIKV during pregnancy show no apparent problems at birth but develop post-natal microcephaly or other neurological alterations [24,25]. This has been seen in symptomatic and asymptomatic mothers [26], which confirms the need for follow-ups for these children with an opportune intervention. The newborns in this study did not show any characteristics associated with ZIKV infection during pregnancy, but the data available suggest that newborns born to mothers who were infected with ZIKV at some point during pregnancy may have low birth weight and height or be born prematurely, these are characteristics described for CZS [7,8]. In addition, when we analyzed these characteristics in a multivariate analysis, we found in a model adjusted to other variables like maternal obstetric risk age and preeclampsia/eclampsia, ZIKV-positive cases did not show a significant association as a risk factor for the development of low weight at birth and low size in the newborns. These data suggest the need to carry out more studies with greater statistical power as well as follow-ups for the newborns of mothers infected with ZIKV during pregnancy. On the other hand, preeclampsia/eclampsia was identified in our study as a significant risk factor for low birth weight; this finding highlights the need for early identification of pregnant women with these conditions because of the possible alterations that could develop in the newborns, and that could synergistically add to ZIKV infection. Consistent with this, it has been documented that ZIKV replicates in endothelial cells [27], which could be a risk factor for preeclampsia/eclampsia [28].

The analyzed data in this work are solely from positive ZIKV cases during the second and third pregnancy trimester of the pregnancy. The possibility of the women having been infected for a considerable time before the samples were obtained is not discarded due to the fact that the participants are asymptomatic. It is difficult to know their previous period of exposure to the virus.

Some viruses cause an illness, but later, the immunological system eliminates them from the system. These kinds of infections are known as acute infections [29]. However, some infections can be persistent depending on the replicative mechanisms of the virus and some characteristics of the host. In these cases, the host immune system does not completely eliminate the virus, and it can remain for a long period of time [30]. The persistence of these viruses in human fluids and tissue is associated with the development of illnesses that should be major targets to be addressed by public health authorities. Whereas viruses with DNA genome (such as hepatitis B, Epstein–Barr, and Type 1 Herpes Simplex) are some viruses that persist and cause long-term health issues [31], it is also known that viruses with RNA genome can cause persistence too, such as Human Immunodeficiency Virus (HIV) and Hepatitis C (HCV), both with great health repercussions [32]. Recent studies suggest that ZIKV persists in different parts of the human body because of the presence of permissive cells; this has been observed in vivo and in vitro studies [33,34,35]. The described anatomical sites for ZIKV persistence are the nervous system and the sexual tract for both males and females [36,37,38,39,40,41]. In the case of pregnant women and symptomatic patients with comorbidities, it has been found that ZIKV can remain in the bloodstream for a prolonged period of time. The longest-reported viremia for a symptomatic person was a man who presented comorbidities and for whom it lasted for 275 days [42]. In this work, we report the case of a woman in whom the presence of ZIKV was detected 90 days after delivery and is, to our knowledge, the longest case registered in the literature in asymptomatic pregnant women. This female patient presented health problems, but in the study, whether the persistence of ZIKV was related to any sign or symptom was not analyzed. A prolonged viremia may be related to increased damage, but more research is needed. The data obtained in this study suggest the need for a follow-up for ZIKV persistence in the bloodstream in patients with comorbidities and pregnant women. A long viremia may have a bigger impact on the health of the patient, and it could also increase the dissemination of the virus to the vector which translates into a high epidemiologic impact.

Limitations of this study include the sample size and the impossibility of accessing the complete file information because the medical records did not contain all the variables analyzed. However, the fact that ZIKV was found in 4.4% of the sample suggests the need for a change of strategy in the study of asymptomatic patients and in the pregnancy control recommendations for women. As of today, only symptomatic women infected with ZIKV are being studied. Also, the identified risks for newborn babies do not show any statistical relevance but the problems derived from those risks do have a clinical significance because of the impact in the life of the newborn. Due to the potential ailments that can appear in the newborn at birth or later in life, it is recommended that ZIKV detection should be included in the basic set of tests performed for pregnant women in endemic zones. More studies that focus on health issues triggered by viruses causing persistence and teratogenic problems are urgently needed so health authorities can have valuable scientific knowledge that can support decision-making.

## Figures and Tables

**Table 1:** General characteristics of asymptomatic pregnant women according to the result of RT-PCR to ZIKV.

Characteristics	Positives to ZIKV *n* = 5	Negatives to ZIKV *n* = 109
Age (years), Mean ± SD	25.4 ± (8.2)	24.9 ± (6.5)
Weight (kg), Mean ± SD	77.4 ± (14.3)	72.1 ± (14.2)
Size (cm), Mean ± SD	162.8 ± (7.9)	156.1 ± (5.5)
Nationality, *n* (%)		
Mexican	5 (100%)	107 (98.2%)
Honduran	0 (0%)	1 (0.9%)
Colombian	0 (0%)	1 (0.9%)
**Gynecological–Obstetric Variable**		
Abortions, *n* (%)	0 (0%)	1 (0.9%)

SD: Standard deviation. RT-PCR: Reverse-Transcription Polymerase Chain Reaction. ZIKV: Zika Virus.

**Table 2:** Detection of ZIKV in serum of asymptomatic pregnant women.

Institution	Samples (Positives)	Prevalence	95% CI
Total Population (GHT + HSHV)	114 (5)	4.4%	1.4–9.9%
GHT	88 (3)	3.4%	0.7–9.6%
HSHV	26 (2)	7.7%	0.9–25.1%

ZIKV: Zika Virus. GHT: General Hospital Tarimoya. HSHV: High Specialty Hospital of Veracruz. 95% CI: 95% confidence interval obtained by exact binomial test.

**Table 3:** Risk of alterations in newborns of asymptomatic pregnant women to ZIKV.

Cases (Asymptomatic Pregnant Women)	With the Event of Interest at Birth	Without the Event of Interest at Birth	Relative Risk	95% CI	*p*-Value
ZIKV Result	Low Weight *n* = 6	Normal Weight *n* = 49		1.6–24.8	0.055
Positive, *n* = 4	2 (50.0%)	2 (50.0%)	6.4		
Negative, *n* = 51 *	4 (7.8%)	47 (92.2%)	1		
ZIKV Result	Low Size *n* = 8	Normal Size *n* = 44		1.2–13.7	0.107
Positive, *n* = 4	2 (50.0%)	2 (50%)	4.0		
Negative, *n* = 48 *	6 (12.5%)	42 (87.5%)	1		
ZIKV Result	With Prematurity *n* = 9	Without Prematurity *n* = 46		1.1–12.1	0.121
Positive, *n* = 4	2 (50.0%)	2 (50%)	3.6		
Negative, *n* = 51 *	7 (13.7%)	44 (86.3%)	1		
ZIKV Result	Altered Head Circumference *n* = 8	Normal Head Circumference *n* = 38	NA	NA	1.000
Positive, *n* = 4	0 (0.0%)	4 (100%)			
Negative, *n* = 42 *	8 (19.1%)	34 (80.9%)			

*n*: Number of subjects. ZIKV: Zika Virus. 95% CI: 95% confidence interval obtained by exact binomial test. * Subjects whose files did not contain information on the event of interest were discarded from this analysis. The reference group is indicated by an RR = 1. *p* value obtained by Fisher’s chi-square or exact test. NA: Not applicable. Low birth weight was defined as less than 2500 g at birth, according to criteria outlined by the World Health Organization and the National Federation of Neonatology in Mexico City. Low birth size was defined according to guidelines for surveillance in environments with risk of ZIKV circulation. Prematurity was defined as those infants with a gestational age of less than 37 weeks, according to criteria indicated by the World Health Organization and the National Federation of Neonatology in Mexico A.C. Altered cephalic perimeter was defined as established by the guidelines for surveillance in environments with risk of ZIKV.

**Table 4:** Multivariate analysis of the association of a history of asymptomatic ZIKV infection with low weight, low size, and prematurity at birth of the newborn.

	Model 1		Model 2		Model 3	
	OR (95% CI)	*p*-Value	OR (95% CI)	*p*-Value	OR (95% CI)	*p*-Value
Outcome: Low Weight at Birth						
Positive to RT-PCR ZIKV	11.8 (1.3–107.1)	**0.03**	11.7 (1.1–121.6)	**0.04**	4.8 (0.3–73.4)	0.26
Obstetric Risk Age *	---	---	4.1 (0.6–28.0)	0.15	3.7 (0.5–30.0)	0.23
Preeclampsia/Eclampsia	---	---	---	---	13.2 (1.3–137.6)	**0.03**
Outcome: Low Size at Birth						
Positive to RT-PCR ZIKV	7.0 (0.8–59.4)	0.08	6.7 (0.7–62.7)	0.10	10.3 (0.7–156.5)	0.09
Obstetric Risk Age *	---	---	3.5 (0.7–17.6)	0.13	3.8 (0.7–19.7)	0.12
Preeclampsia/Eclampsia	---	---	---	---	0.4 (0.02–9.0)	0.56
Outcome: Prematurity						
Positive to RT-PCR ZIKV	6.3 (0.8–52.2)	0.09	6.1 (0.6–58.8)	0.12	2.9 (0.2–36.9)	0.42
Obstetric Risk Age *	---	---	4.5 (0.9–21.7)	0.06	4.1 (0.8–21.2)	0.09
Preeclampsia/Eclampsia	---	---	---	---	6.7 (0.7–62.3)	0.09

OR: Odds ratio calculated by binary logistic regression. Method of introducing variables: “Enter” in three blocks. 95% CI: 95% confidence interval. RT-PCR ZIKV: Reverse-Transcription Polymerase Chain Reaction for Zika Virus. Model 1: Without adjustment Model 2: Age-adjusted for obstetric risk. Model 3: Age-adjusted for obstetric risk and preeclampsia/eclampsia. * Obstetric risk age: <20 years or >35 years of age.

**Table 5:** Trimester of pregnancy in which ZIKV is detected.

RT-PCR to ZIKV Test Result	1° Trimester	2° Trimester	3° Trimester	No Information	Total
Positive Cases	0 (0%)	1 (20%)	4 (80%)	0 (0%)	5 (100%)
Negatives Cases	7 (6.4%)	22 (20.2%)	72 (66.1%)	8 (7.3%)	109 (100%)
Total	7 (6.1%)	23 (20.2%)	76 (66.7%)	8 (7.0%)	114 (100%)

RT-PCR: Reverse-Transcription Polymerase Chain Reaction. ZIKV: Zika Virus.

## Data Availability

The original contributions presented in this study are included in the article. Further inquiries can be directed to the corresponding author.

## References

[1] Dick G.W., Kitchen S.F., Haddow A.J. (1952). Isolations and serological specificity. Trans. R. Soc. Trop. Med. Hyg..

[2] Rawal G., Yadav S., Kumar R. (2016). Zika virus: An overview. J. Fam. Med. Prim. Care.

[3] Duffy M.R., Chen T.H., Hancock W.T., Powers A.M., Kool J.L., Lanciotti R.S., Pretrick M., Marfel M., Holzbauer S., Dubray C. (2009). Zika virus outbreak on Yap Island, Federated States of Micronesia. N. Engl. J. Med..

[4] Equihua M., Ibáñez-Bernal S., Benítez G., Estrada-Contreras I., Sandoval-Ruiz C.A., Mendoza-Palmero F.S. (2017). Establishment of *Aedes aegypti* (L.) in mountainous regions in Mexico: Increasing number of population at risk of mosquito-borne disease and future climate conditions. Acta Trop..

[5] SINAVE/DGE/SS DdVEdET Casos Confirmados Autóctonos de Enfermedad por Virus del Zika por Entidad Federativa 2024 [“Casos Confirmados de Enfermedad por Virus del Zika”, Semana Epidemiológica 39 del 2024]. https://www.gob.mx/cms/uploads/attachment/file/948032/CuadroCasosZikayEmbsem39_2024.pdf.

[6] Moore C.A., Staples J.E., Dobyns W.B., Pessoa A., Ventura C.V., Da Fonseca E.B., Ribeiro E.M., Ventura L.O., Neto N.N., Arena J.F. (2017). Characterizing the Pattern of Anomalies in Congenital Zika Syndrome for Pediatric Clinicians. JAMA Pediatr..

[7] Da Silva Pastich Gonçalves F.C.L., De Carvalho Lima M., De Alencar Ximenes R.A., De Barros Miranda-Filho D., Martelli C.M.T., Rodrigues L.C., De Souza W.V., De Lira P.I.C., Eickmann S.H., Araújo T.V.B. (2021). A new insight into the definition of microcephaly in Zika congenital syndrome era. Cad. De Saúde Pública.

[8] Cooper H.J., Iwamoto M., Lash M., Conners E.E., Paladini M., Slavinski S., Fine A.D., Kennedy J., Heinke D., Ciaranello A. (2019). Maternal Zika Virus Infection: Association With Small-for-Gestational-Age Neonates and Preterm Birth. Obstet. Ginecology.

[9] Van der Linden V., de Lima Petribu N.C., Pessoa A., Faquini I., Paciorkowski A.R., Van Der Linden H., Silveira-Moriyama L., Cordeiro M.T., Hazin A.N., Barkovich A.J. (2019). Association of Severe Hydrocephalus with Congenital Zika Syndrome. JAMA Neurol..

[10] Mulkey S.B., Arroyave-Wessel M., Peyton C., Bulas D.I., Fourzali Y., Jiang J., Russo S., McCarter R., Msall M.E., Du Plessis A.J. (2020). Neurodevelopmental Abnormalities in Children Within Utero Zika Virus Exposure Without Congenital Zika Syndrome. JAMA Pediatr..

[11] Martinot A.J., Abbink P., Afacan O., Prohl A.K., Bronson R., Hecht J.L., Borducchi E.N., Larocca R.A., Peterson R.L., Rinaldi W. (2018). Fetal Neuropathology in Zika Virus-Infected Pregnant Female Rhesus Monkeys. Cell.

[12] Meaney-Delman D., Oduyebo T., Polen K.N., White J.L., Bingham A.M., Slavinski S.A., Heberlein-Larson L., St George K., Rakeman J.L., Hills S. (2016). Prolonged Detection of Zika Virus RNA in Pregnant Women. Obstet. Gynecol..

[13] Rodó C., Suy A., Sulleiro E., Soriano-Arandes A., Maiz N., García-Ruiz I., Arévalo S., Rando A., Anton A., Méndez V. (2019). Pregnancy outcomes after maternal Zika virus infection in a non-endemic region: Prospective cohort study. Clin. Microbiol. Infect..

[14] Suy A., Sulleiro E., Rodo C., Vázquez É., Bocanegra C., Molina I., Esperalba J., Sánchez-Seco M.P., Boix H., Pumarola T. (2016). Prolonged Zika Virus Viremia during Pregnancy. N. Engl. J. Med..

[15] DGE/DGAE/InDRE/INPer (2018). Casos confirmados de Síndrome Congénito asociado a Zika, México 2016-2018 Secretaría de Salud: Secretaría de Salud. https://www.gob.mx/cms/uploads/attachment/file/534151/Cuadro_Sx_Congenito_asociado_a_Zika.pdf.

[16] Haby M.M., Pinart M., Elias V., Reveiz L. (2018). Prevalence of asymptomatic Zika virus infection: A systematic review. Bull. World Health Organ..

[17] Komarasamy T.V., Adnan N.A.A., James W., Balasubramaniam V.R.M.T. (2022). Zika Virus Neuropathogenesis: The Different Brain Cells, Host Factors and Mechanisms Involved. Front. Immunol..

[18] Weilg C., Troyes L., Villegas Z., Silva-Caso W., Mazulis F., Febres A., Troyes M., Aguilar-Luis M.A., Del Valle-Mendoza J. (2018). Detection of Zika virus infection among asymptomatic pregnant women in the North of Peru. BMC Res. Notes.

[19] Branco R.C.C., Brasil P., Araujo J.M.G., Cardoso F.O., Batista Z.S., Leitão V.M.S., Da Silva M.A.C.N., De Castro L.O., Valverde J.G., Jeronimo S.M.B. (2021). Evidence of Zika virus circulation in asymptomatic pregnant women in Northeast, Brazil. PLoS Negl. Trop. Dis..

[20] Calvet G.A., Kara E.O., Giozza S.P., Bôtto-Menezes C.H.A., Gaillard P., De Oliveira Franca R.F., De Lacerda M.V.G., Da Costa Castilho M., Brasil P., De Sequeira P.C. (2018). Study on the persistence of Zika virus (ZIKV) in body fluids of patients with ZIKV infection in Brazil. BMC Infect. Dis..

[21] Aagaard K.M., Lahon A., Suter M.A., Arya R.P., Seferovic M.D., Vogt M.B., Hu M., Stossi F., Mancini M.A., Harris R.A. (2017). Primary Human Placental Trophoblasts are Permissive for Zika Virus (ZIKV) Replication. Sci. Rep..

[22] Schwartz D.A. (2017). The Origins and Emergence of Zika Virus, the Newest TORCH Infection: What’s Old Is New Again. Arch. Pathol. Lab. Med..

[23] Reynolds M.R., Jones A.M., Petersen E.E., Lee E.H., Rice M.E., Bingham A., Ellington S.R., Evert N., Reagan-Steiner S., Oduyebo T. (2017). Zika Pregnancy Registry Collaboration. Vital Signs: Update on Zika Virus -Associated Birth Defects and Evaluation of All U.S. Infants with Congenital Zika Virus Exposure—U.S. Zika Pregnancy Registry, 2016.

[24] Van der Linden V., Pessoa A., Dobyns W., Barkovich A.J., Van Der Linden Júnior H., Filho E.L.R., Ribeiro E.M., De Carvalho Leal M., De Araújo Coimbra P.P., De Fátima Viana Vasco Aragão M. (2016). Description of 13 Infants Born During October 2015–January 2016 with Congenital Zika Virus Infection Without Microcephaly at Birth—Brazil.

[25] Aragao M., Holanda A.C., Brainer-Lima A.M., Petribu N.C.L., Castillo M., Van Der Linden V., Serpa S.C., Tenório A.G., Travassos P.T.C., Cordeiro M.T. (2017). Nonmicrocephalic Infants with Congenital Zika Syndrome Suspected Only after Neuroimaging Evaluation Compared with Those with Microcephaly at Birth and Postnatally: How Large Is the Zika Virus “Iceberg”?. AJNR Am. J. Neuroradiol..

[26] Chimelli L., Moura Pone S., Avvad-Portari E., Vasconcelos Z.F.M., Zin A.A., Cunha D.P., Thompson N.R., Moreira M.E.L., Wiley C.A., Da Silva Pone M.V. (2018). Persistence of Zika Virus After Birth: Clinical, Virological, Neuroimaging, and Neuropathological Documentation in a 5-Month Infant With Congenital Zika Syndrome. J. Neuropathol. Exp. Neurol..

[27] Gandolfo C., Terrosi C., Prathyumnan S., Anichini G., Savellini G.G., Morgante G., Cusi M.G. (2022). Human Polymorphonuclear Cells Support Zika Virus to Cross Endothelial Monolayer and Access Bloodstream. Pathogens.

[28] Nieves C., da Costa-Ghignatti P.V., Aji N., Bertagnolli M. (2024). Immune Cells and Infectious Diseases in Preeclampsia Susceptibility. Can. J. Cardiol..

[29] Vianna R.A.O., Rua E.C., Fernandes A.R., dos Santos T.C.S., Dalcastel L.A.B., dos Santos M.L.B., Paula P.d.S.d., de Carvalho F.R., Faria A.d.O.P.d., Almeida P.L. (2020). Experience in diagnosing congenital Zika syndrome in Brazilian children born to asymptomatic mothers. Acta Trop..

[30] Flint S.J., Racaniello V.R., Skalka A.M. (2009). Principles of Virology. Molecular Biology, Pathogenesis, and Control of Animal Viruses.

[31] Knipe D.M., Howley P.M. (2007). Fields Virology.

[32] Boldogh I., Albrecht T., Porter D.D., Baron S. (1996). Medical Microbiology.

[33] McCarthy M.K., Morrison T.E. (2017). Persistent RNA virus infections: Do PAMPS drive chronic disease?. Curr. Opin. Virol..

[34] Miner J.J., Diamond M.S. (2017). Zika Virus Pathogenesis and Tissue Tropism. Cell Host Microbe.

[35] Pagani I., Ghezzi S., Ulisse A., Rubio A., Turrini F., Garavaglia E., Candiani M., Castilletti C., Ippolito G., Poli G. (2017). Human Endometrial Stromal Cells Are Highly Permissive To Productive Infection by Zika Virus. Sci. Rep..

[36] Da Silva I.R.F., Frontera J.A., Bispo de Filippis A.M., Nascimento O., RIO-GBS-ZIKV Research Group (2017). Neurologic Complications Associated With the Zika Virus in Brazilian Adults. JAMA Neurol..

[37] Salam A.P., Horby P.W. (2017). The Breadth of Viruses in Human Semen. Emerg. Infect. Dis..

[38] Atkinson B., Hearn P., Afrough B., Lumley S., Carter D., Aarons E.J., Simpson A.J., Brooks T.J., Hewson R. (2016). Detection of Zika Virus in Semen. Emerg. Infect. Dis..

[39] Nicastri E., Castilletti C., Liuzzi G., Iannetta M., Capobianchi M.R., Ippolito G. (2016). Persistent detection of Zika virus RNA in semen for six months after symptom onset in a traveller returning from Haiti to Italy, February 2016. Euro Surveill..

[40] Prisant N., Bujan L., Benichou H., Hayot P.-H., Pavili L., Lurel S., Herrmann C., Janky E., Joguet G. (2016). Zika virus in the female genital tract. Lancet Infect. Dis..

[41] Mehta R., Soares C.N., Medialdea-Carrera R., Ellul M., da Silva M.T.T., Rosala-Hallas A., Jardim M.R., Burnside G., Pamplona L., Bhojak M. (2018). The spectrum of neurological disease associated with Zika and chikungunya viruses in adults in Rio de Janeiro, Brazil: A case series. PLoS Negl. Trop. Dis..

[42] Silva K.R., Bica B., Pimenta E.S., Serafim R.B., Abreu M.M., Gonçalves J.L.S., Santana L.D.S., Cabral-Castro M.J., Peralta J.M., Cavalcanti M.G. (2018). Fatal Human Case of Zika and Chikungunya Virus Co-Infection with Prolonged Viremia and Viruria. Diseases.

